# From burden to backbone: the regenerative potential of food waste through digital, biological, and technological innovation

**DOI:** 10.3389/fnut.2025.1675732

**Published:** 2025-09-24

**Authors:** Francesca Girotto, Giovanni Beggio

**Affiliations:** ^1^Department of Environmental Science and Policy, Università degli Studi di Milano, Milan, Italy; ^2^Department of Civil, Environmental and Architectural Engineering, University of Padova, Padua, Italy

**Keywords:** regenerative circular bioeconomy, upcycling, digital enablers, microbial electrochemical systems, solar photoreforming, green extraction methods, biocomposites, biomedical upcycling

## Abstract

This perspective article outlines a cross-sectoral roadmap that leverages digital, biological, and material innovations to transform unavoidable food and beverage organic waste into high-value resources. In this manuscript, the term “regenerative circular bioeconomy” refers to a systemic approach that not only minimises waste and closes resource loops but also enhances the resilience of natural and social systems. “Upcycling” is here defined as the transformation of organic residues into products of higher functional or economic value compared to their original use. “Digital enablers” are considered as data-driven tools, such as artificial intelligence, machine learning, and digital twins, which support the optimisation and monitoring of valorisation processes. Artificial intelligence is positioned as a systemic enabler of real-time diagnostics, redistribution, and forecasting, supporting both the quantification and reduction of organic waste. In parallel, the integration of mathematical modelling with digital technologies is increasingly driving the development of data-driven algorithms aimed at optimising process conditions for upcycling strategies within valorisation pathways. Regarding traditional recovery routes, the article highlights frontier technologies including microbial electrochemical systems, solar photoreforming, and green extraction methods. It also presents cutting-edge applications such as the use of organic waste in biocomposites and the emerging biomedical upcycling of slaughterhouse by-products for tissue engineering. Through this interdisciplinary lens, the article advocates for a regenerative circular bioeconomy supported by infrastructural investment, ethical governance, and comprehensive life-cycle validation.

## Graphical abstract

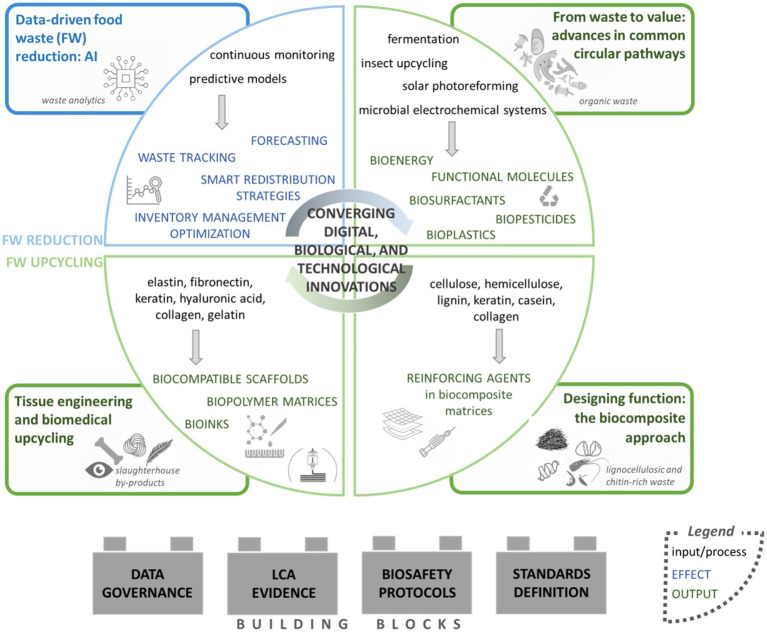

## Introduction

The food and beverage sector is increasingly embracing open innovation strategies to improve environmental performance and implement more sustainable sourcing practices. While these efforts are essential, they are not sufficient to eliminate waste generation, particularly organic waste, which arises at multiple stages across the supply chain. From on-farm losses during cultivation and harvesting to quality-based discards during sorting and processing, and spoilage throughout transport and storage, organic waste is a structurally embedded outcome of handling perishable and biologically variable materials.

The strong nexus between food waste and climate change is well documented, with food waste alone contributing an estimated 8–10% of global greenhouse gas emissions (1). This has led to urgent calls for cross-sectoral engagement, anchored in the United Nations’ Sustainable Development Goals, with Goal 12.3 being the most relevant to this context, aiming to halve per capita food waste and reduce food losses across the supply chain by 2030.

Yet to realise this ambition, efforts must be grounded in a deeper and more systemic understanding of the issue’s scale, distribution, and environmental burden. While a share of this waste can be reduced through process optimisation and technological innovation, organic waste is fundamentally unavoidable. In an era of accelerating environmental change, the sustainable management of organic residues must be recognised not only as an industrial necessity, but as a global environmental priority. This Perspective highlights a selection of forward-looking strategies illustrating how science, technology, and policy can converge to reduce and transform organic residues into regenerative resources.

## Measuring food waste: beyond what reaches the plate

According to the 2024 UNEP Food Waste Index Report, an estimated 1.05 billion tonnes of food were wasted globally in 2022. Of this total, 131 million tonnes (12%) originated from retail, 290 million tonnes (28%) from food services, and a staggering 631 million tonnes (60%) from households. On a per capita basis, this corresponds to approximately 132 kg of food waste per person each year, with 79 kg occurring in domestic kitchens alone. These figures highlight the persistent need to raise public awareness and foster a culture of responsible consumption, a low-cost, high-impact intervention that remains consistently undervalued. However, it is important to note that these estimates focus primarily on post-harvest and consumption stages, excluding a vast layer of upstream food losses that occur during agricultural production and industrial processing. Climatic disruptions, pest infestations, harvesting inefficiencies, and outdated equipment routinely lead to the loss of large quantities of edible biomass, losses rarely reflected in national food waste statistics.

When food is wasted, it is not only the end product that is lost, but also all the embedded resources required to produce it, particularly water, energy, and labour. This inefficiency places a considerable economic burden on the entire supply chain and represents a misuse of increasingly scarce natural inputs. Agriculture alone accounts for nearly 70% of global freshwater withdrawals (2), making upstream losses highly detrimental. The situation is further compounded by projections from the United Nations, which warn of a 40% global freshwater shortfall by 2030, underscoring the urgent need to reduce waste and improve resource-use efficiency across the agri-food system.

## Artificial intelligence: unlocking data-driven waste reduction

Artificial Intelligence (AI) is emerging as a transformative enabler in the quest to reduce food and beverage waste by identifying inefficiencies across all stages of the supply chain. While traditional lean tools, such as Value Stream Mapping and the Pareto principle, have been widely used for the expert-driven diagnostics of key factors contributing to the majority of waste (3), AI extends these approaches by providing real-time, scalable, and automated analyses. AI-powered systems are increasingly deployed in large-scale operations to enhance forecasting, optimise inventory management, improve waste tracking, and guide smart redistribution strategies (4). A key advantage lies in the ability of these systems to identify waste hotspots by analysing both real-time and historical data, thereby enabling the development of predictive models. Furthermore, AI enhances cold-chain logistics and shelf-life monitoring, two elements critical for minimizing spoilage in perishable goods. However, widespread adoption is still hindered by fragmented data infrastructures, elevated costs, and uneven digital readiness, especially among small and medium-sized enterprises, requiring stronger cross-sectoral collaboration and strategic investment in digital infrastructure and workforce training (5).

Institutional settings, such as hospitals, school canteens, universities, and airports, represent critical nodes in the foodservice sector where organic waste is often substantial due to rigid menu planning, low forecasting accuracy, and operational constraints. These environments can greatly benefit from AI-enabled solutions. In particular, smart bin systems equipped with integrated sensors are increasingly being piloted across institutional foodservice environments in Europe and North America, including cafeterias and hospital kitchens, where they enable continuous monitoring and quantification of food waste. When combined with historical consumption data, these tools can support evidence-based adjustments to portion sizing, menu planning, and procurement. As reported by Yip et al. (6), hospital foodservices have already achieved notable reductions in waste-related emissions through structured donation programs. Still grounded in traditional approaches, these efforts could be strengthened by intelligent forecasting and tracking, streamlining digital redistribution.

Across all operational contexts, however, the path to implementation remains complex. The deployment of digital analytics platforms is constrained not only by technological and financial barriers but also by ethical considerations related to data governance and potential job displacement. AI does not represent a plug-and-play solution; it requires robust infrastructure, cross-sectoral alignment, and policy support to ensure tangible, scalable reductions in food waste.

## From waste to value: advances in traditional valorisation routes

While reducing waste generation remains a priority, equal attention should also be directed at how unavoidable residues are managed and valorised. To move beyond containment, we must reimagine organic waste as a source of value that can fuel regenerative innovation (Table 1). Historically, food waste recovery has centred on bioenergy generation, including biogas, bio-methane, biodiesel, ethanol, and, more recently, hydrogen.

**Table 1:** Comparative overview of selected emerging valorisation technologies.

Technology	Advantages	Limitations	Readiness level
Microbial electrochemical systems (METs)	Dual recovery of energy carriers and value-added products; potential for hydrogen/electricity generation	Biofilm instability; low yields; challenges in scaling reactor designs	Early pilot (TRL 4–5)
Solar photoreforming	Clean hydrogen and carbon by-products; sunlight-driven; dual output (fuel + solid carbonaceous co-products)	Requires substrate pre-treatment; technology still at laboratory scale	Lab/pilot (TRL 3–4)
Fermentation (solid-state, submerged)	Production of bioproducts (biosurfactants, bioplastics precursors, biopesticides); scalable with optimised design	Critical strain selection; challenging process control; wastewater management	Pilot to industrial (TRL 6–9)
Insect upcycling (e.g., by *Hermetia illucens*)	Efficient conversion of heterogeneous substrates; production of protein-rich and fat-rich biomass; multiple end uses (feed, biodiesel, chitin/chitosan, peptides)	Variability in nutrient content and feedstock quality; regulatory fragmentation for feed applications; risk of contaminants	Pilot to industrial (TRL 6–9)
Green extraction (microwave, ultrasound, pulsed electric field)	Higher yields; reduced solvent use; preservation of bioactivity	Residual biomass management; equipment and operational costs	Pilot to industrial [TRL 5–9 depending on technology (PEF less mature)]

Microbial electrochemical technologies (METs) offer an innovative way to recover both energy and valuable resources from solubilized organic waste (7). These systems use electroactive bacteria that naturally transfer electrons to electrodes while breaking down organic compounds. As a result, METs have the potential to generate electricity or hydrogen from pre-treated organic fractions of food waste, although current yields remain limited by scale-up challenges and suboptimal system efficiencies, partly due to biofilm instability issues (8). An emerging frontier in waste-to-energy valorisation is solar reforming, a clean, sunlight-driven process that converts organic waste into hydrogen fuel and solid carbon materials (9). This photoreforming technology uses solar-active catalysts, often immobilised on floating supports, to break down organic compounds. When applied to food waste, pre-treatment steps such as homogenisation, enzymatic hydrolysis, or alkaline solubilisation are typically required to convert complex residues (e.g., carbohydrates, lipids, and proteins) into aqueous substrates suitable for light-driven reactions (10). Upon solar irradiation, photoreforming generates hydrogen gas while simultaneously producing solid carbon residues, which can be further engineered into advanced carbon-based materials, such as precursors for green photocatalysts for environmental or energy applications (11, 12). This dual-output approach offers a sustainable route to clean energy and carbon valorisation, minimising reliance on energy-intensive processes and fossil-derived reagents. Both METs and solar reforming are particularly suited for the valorisation of liquid effluents generated by food and beverage industries, where organic compounds are already solubilised or can be easily pre-treated into aqueous substrates amenable to bioelectrochemical or photoreforming conversion. However, despite their promise, both processes remain at a pre-commercial stage. As highlighted by Alvarez-Pugliese et al. (8), the lack of standardised performance metrics, including current efficiency and full cost assessments, underscores the pressing need for scalable reactor designs and techno-economic validation.

Waste-to-energy circular pathways represent only a fraction of a broader and increasingly diversified solution space. A paradigm shift is underway toward multi-purpose circular economy frameworks that recover value from organic waste through integrated strategies. Biosurfactants, biopesticides, and bioplastics, or, more precisely, their molecular building blocks, are valuable bioproducts that can be obtained through fermentative processes of organic waste (13). While submerged fermentation allows for better control of process parameters and is more readily scalable for industrial applications, solid-state fermentation offers several advantages, including cost-effectiveness, lower water requirements, and reduced wastewater generation (14). In both cases, key challenges include the development of robust reactor designs and the careful selection of microbial strains or consortia.

Insect-based upcycling is gaining traction as a promising approach, particularly as regulatory frameworks continue to evolve and shape its large-scale implementation. Larvae, such as *Hermetia illucens* (known as black soldier fly), can convert heterogeneous organic substrates into a protein-rich (up to 45%) and fat-rich (up to 35%) biomass (15) which can be used for the production of animal feed, biodiesel, biofertilizers, or for the extraction of high-value compounds such as antimicrobial peptides, chitin, and chitosan (16). However, variations in nutrient content, moisture levels, and the presence of contaminants in the feedstock can significantly affect larval yield and biomass quality. Additionally, when targeting feed applications, the lack of harmonized regulatory frameworks across countries represents a major barrier to large-scale deployment (16).

The extraction of functional molecules from organic waste represents another relevant valorisation pathway. These extend beyond well-known polyphenols and flavonoids to include pectins, dietary fibres, inositol, oligosaccharides, proteins, and structural carbohydrates, compounds of growing relevance in functional food, nutraceutical, cosmetic, and pharmaceutical sectors (15, 17). Yet, conventional solvent-based extraction techniques remain problematic due to their reliance on toxic reagents and the generation of hazardous effluents. In contrast, green extraction technologies, such as microwave-assisted [e.g., as optimised for ursolic acid from apple pomace (18)], ultrasound-assisted, and pulsed electric field methods, offer cleaner, more energy-efficient alternatives that can be paired with safer, less toxic solvents. These not only enhance yields but also help preserve the biochemical integrity and bioactivity of target compounds (15). However, a critical drawback of compound extraction, even via green technologies, is the generation of large volumes of residual biomass to be managed. Valorisation strategies must therefore be designed as part of an integrated cascade approach, ensuring that secondary waste streams are anticipated, quantified, and further valorised.

## Waste-to-material innovation: the rise of biocomposites

Yet, the material valorisation of organic waste is not confined to biochemical pathways, emerging strategies in material engineering are expanding the horizon of what waste can become. In particular, the development of biocomposites leverages organic waste streams as functional fillers, reinforcements, or even matrix components in advanced material systems. This strategy actively contributes to the replacement of fossil-based plastics and synthetic fibres across a range of industrial applications.

Lignocellulosic residues, such as fruit and vegetable peels, pomace, shell flour, husks, wheat straw, and bagasse, are rich in cellulose, hemicellulose, and lignin. These components can be extracted, or alternatively, the waste can be mechanically processed for use as reinforcing agents in polymer or cementitious matrices (19), potentially enhancing both the mechanical strength and end-of-life degradability of the resulting composites (20). Promising applications include sustainable packaging, automotive interior components, construction materials, and 3D-printed prototypes or parts. Sánchez-Safont et al. (21) provided a comprehensive analysis of multicomponent polymer systems incorporating renewable polyesters, such as PLA (polylactic acid) and PBS (polybutylene succinate), with natural fibres and agro-waste fillers. Their review highlights how blending strategies, such as physical mixing or reactive compatibilization, can mitigate the mechanical limitations and brittleness typically associated with biopolyesters. Indeed, bio-based reinforcements from waste substrates face challenges such as a tendency to absorb moisture at room temperature, which can compromise their interfacial bonding with the matrix material (22). Therefore, chemical or physical treatments of natural fibres, as well as incorporation of nanofillers, are crucial to enhance adhesion at the interface (23), which is key to improving mechanical performance, including tensile strength, impact resistance, and thermal stability. A case in point is the work by Aramwit et al. (24), who demonstrated that combining rice husk and coir in gypsum matrices can significantly improve the mechanical, thermal, and acoustic performance of ceiling tiles. Their optimised formulation could not only increase flexural strength and modulus but also improve sound absorption in critical frequency ranges (2,500–4,500 Hz), while reducing thermal conductivity and flammability risks.

Beyond lignocellulosic inputs, proteinaceous by-products, such as feathers, fish scales, and dairy residues, also hold promise as sources of keratin, casein, and collagen, which can serve as functional additives or reinforcing agents in biocomposite matrices. For instance, Europe produces over 3.6 million tonnes of poultry feather waste annually, yet less than 25% is currently valorised (25, 26). A practical and efficient use of these fibres, known for their excellent thermal insulation, sound absorption, and compression resistance (27), is the production of nonwoven mats or preformed panels made entirely of whole feathers, which may serve as sustainable alternatives for semi-structural components in construction (e.g., panels, boards) and vehicle interiors (26).

The feasibility of these biocomposites depends not only on their technical performance but also on market acceptance, industrial scalability, and regulatory clarity. Material variability and the absence of unified standards still represent major barriers to widespread implementation. Moreover, life cycle assessments (LCAs) are essential to validate the environmental benefits of such materials compared to conventional alternatives, especially regarding their end-of-life management.

Overall, by integrating agro-industrial residues into the design of functional materials, biocomposites represent a high-value application that aligns with both waste valorisation and decarbonization goals.

## Tissue engineering and biomedical upcycling: the unexpected frontier

Given the growing shortage of transplantable organs and tissues, recent studies have explored the conversion of slaughterhouse by-products into biomaterials for regenerative medicine, pushing the boundaries of circularity. Among these, keratin-rich waste (e.g., feathers, wool, horns, hooves) is being repurposed to produce biodegradable, biocompatible scaffolds and biopolymer matrices (28). This strategy not only aligns with zero-waste goals but also opens avenues for replacing synthetic polymers in clinical settings (29).

Corridon et al. (30) presented a hypothesis-driven strategy for developing sustainable keratoplasty models by repurposing bovine, porcine, or ovine corneal tissues otherwise discarded during meat processing. The decellularized tissues are intended to be reseeded with urine-derived stem cells (USCs), either directly or following reprogramming into induced pluripotent stem cells (u-iPSCs), to generate compartment-specific corneal structures. This preclinical model aims to support tissue engineering research while exemplifying the principles of circular bioeconomy and biomedical innovation (30). Complementing this conceptual framework, Ali et al. (31) experimentally validated that corneal scaffolds derived from refrigerated slaughterhouse ovine eyes retained key mechanical and optical properties for clinical use, including transparency and tensile strength, after decellularization using a low-toxicity zwitterionic biosurfactant which enabled an over 90% reduction in residual DNA content.

In parallel, 3D bioprinting technologies are rapidly advancing, relying on bioinks enriched with extracellular matrix components such as collagen, elastin, fibronectin, and hyaluronic acid abundant within slaughterhouse waste (32). Tarafdar et al. (29) emphasized that animal-derived collagen and gelatin, obtainable from bones and skin, exhibit high biocompatibility and gelation properties suitable for hydrogel matrices, particularly when combined with alginate or chitosan for enhanced stability. Recent efforts have demonstrated the feasibility of using decellularized extracellular matrix (dECM) as a distinct type of bioink, derived from slaughterhouse organs such as liver, spleen, kidney, and lung, with tunable rheological and crosslinking properties suited for precision tissue reconstruction (33, 34). These dECM bioinks have demonstrated compatibility for skin, vascular, and hepatic tissue engineering applications, and could be adapted to incorporate cell-laden hydrogels or growth factors for advanced regenerative protocols.

Such applications redefine the value hierarchy of organic waste, positioning regenerative medicine as a high-value pathway within a broader vision of circular bioeconomy. However, challenges remain in the form of cross-species immunogenicity, consistency in ECM isolation protocols, and regulatory clarity for clinical-grade biomaterials (33).

## Conclusion: from biomass burden to regenerative potential

The sustainable management of organic food and beverage waste cannot rely solely on downstream interventions. It requires a holistic, cross-disciplinary approach that connects food systems, environmental science, biotechnology, and industrial design. As shown, there are diverse opportunities to convert residues into resources. Yet unlocking this potential demands more than innovation: it calls for enabling policy frameworks, multi-sector collaboration, and investment in infrastructure and digital capacity (Table 2). In low- and middle-income countries, tailored financing and targeted capacity-building are essential to scale impactful solutions.

**Table 2:** Key barriers and ethical considerations across circular pathways.

Valorisation domain	Main barriers (economic, regulatory, technical)	Ethical and social considerations
Digital enablers (AI, data-driven tools)	High implementation costs; fragmented data infrastructures; limited digital readiness of SMEs	Data governance, privacy, and potential reconfiguration of workforce roles
Biological/biochemical pathways (fermentation, insect upcycling, green extraction)	Feedstock variability; lack of harmonised regulatory frameworks; uncertainties in techno-economic feasibility	Acceptance of novel bioproducts in food/feed chains; transparency in risk communication; need for transparent sustainability evidence (e.g., social and conventional LCA)
Electrochemical/photocatalytic pathways (METs, solar reforming)	Biofilm instability; low energy yields; pre-treatment requirements; scale-up limitations	Public perception of advanced waste-to-fuel/material processes; need for transparent sustainability evidence (e.g., social and conventional LCA)
Material valorisation (biocomposites)	Absence of unified material standards; limited industrial scalability; market uncertainty on cost competitiveness	Consumer acceptance of waste-derived materials; verification of environmental claims (e.g., via LCA)
Biomedical upcycling (tissue engineering)	Cross-species immunogenicity; variability in biomaterial quality; unclear clinical regulatory pathways	Ethical sourcing of animal by-products; societal perceptions of biomedical reuse of waste; robust ethical and biosafety assessments

Equally important is the incorporation of robust LCAs and cost–benefit analyses to evaluate the actual environmental and economic trade-offs of different valorisation routes, including comparisons between purified extracts and the use of whole raw residues, thereby ensuring that circular strategies are both scalable and robustly sustainable. A more systematic integration of LCA is also urgently needed to identify unintended environmental burdens, such as secondary emissions or residual biomass generation. Expanding the evidence base in this area will be key to guiding both policy and research priorities.

To harness the potential of organic waste valorisation, we must embrace systemic thinking, foster responsible innovation, and invest in both physical and digital infrastructures needed to close the loop, transforming waste into a cornerstone of the regenerative bioeconomy, where digital tools will increasingly support the control and optimisation of upcycling process parameters.

## Data Availability

The original contributions presented in the study are included in the article/supplementary material, further inquiries can be directed to the co-author, FG, francesca.girotto@unimi.it.
